# Toxicokinetics and analytical toxicology of the phenmetrazine-derived new psychoactive substance 3,4-methylenedioxyphenmetrazine studied by means of in vitro systems

**DOI:** 10.1007/s00204-025-03965-w

**Published:** 2025-02-04

**Authors:** Matthias D. Kroesen, Tanja M. Gampfer, Lea Wagmann, Markus R. Meyer

**Affiliations:** Department of Experimental and Clinical Toxicology and Pharmacology, Institute of Experimental and Clinical Pharmacology and Toxicology, Center for Molecular Signaling (PZMS), Saarland University, Homburg, Germany

**Keywords:** 3,4-methylenedioxyphenmetrazine, Analytical toxicology, Toxicokinetics, New psychoactive substance

## Abstract

Compounds derived from known drugs are usually brought on the new psychoactive substance (NPS) market without any previous toxicological risk assessment. The European Union Drugs Agency issued an EU early notification for 3,4-methylenedioxyphenmetrazine (MDPM) in 2024. It is structurally related to the stimulants amphetamine, 3,4-methylenedioxymethamphetamine (MDMA), and phenmetrazine and expected to have similar effects. So far, no scientific reports are available describing its toxicokinetic and analytical profile. This study aimed to provide such data to allow a thorough risk assessment and to ease its analytical detectability in forensic and clinical toxicology and doping control. Data reported include the in vitro plasma protein binding, the in vitro half-life and in vitro metabolism of MDPM by human liver microsomes and S9 fraction (pHLS9) and by HepaRG cells. A monooxygenase mapping and the in vitro cytochrome P450 inhibition of MDPM was elucidated. Results showed that HepaRG cells and pHLS9 formed the same MDPM metabolites via demethylenation and *O*-methylation and that MDPM has a low plasma protein binding and is a low-turnover drug. Monooxygenase mapping revealed that the demethylenation was exclusively CYP2D6-mediated. MDPM showed strong inhibition of CYP2D6 and moderate inhibition of CYP1A2 and CYP3A4. Polymorphisms or the simultaneous intake of substances that are also CYP2D6 substrates can have a considerable impact on the toxicity of MDPM. Based on in vitro data, the demethylenyl-methyl metabolite of MDPM and the parent compound are recommended as analytical urine screening targets.

**Supplementary Information:**

The online version contains supplementary material available at 10.1007/s00204-025-03965-w.

## Introduction

**Fig. 1:**
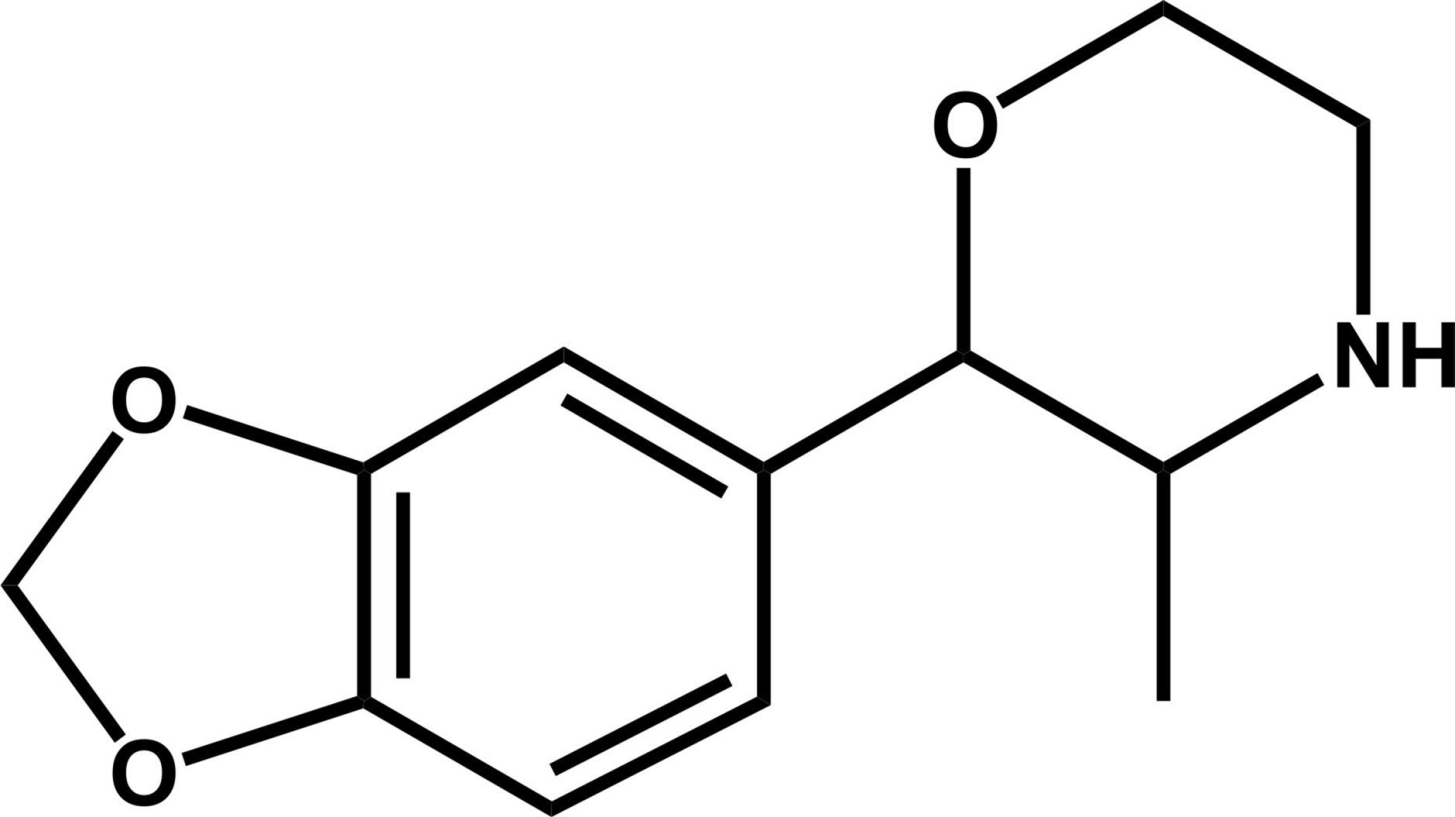
Chemical structure of 3,4-methylenedioxyphenmetrazine (MDPM)

Stimulants are the largest group of new psychoactive substances (NPS) reported to the United Nations Office on Drugs and Crime (UNODC) Early Warning Advisory (UNODC 2024). Their relevance is underlined by rising figures of drug abuse and drug-induced deaths related to synthetic stimulants (EUDA 2023). Surveys of 25 European Union countries conducted between 2016 and 2022 found that 1.3 million young adults in Europe (aged 15 to 34) had used amphetamines in the previous year (EUDA 2023). In general, NPS pose major health risks, as there is rarely any information about toxicity, toxicokinetics, or mode of action. Furthermore, often no data is available on potential drug-drug interactions. In the absence of scientific studies, effects and toxicity are usually only estimated by comparison to similar substances. This represents major challenges for medical professionals, toxicologists and legislation. To keep pace with the advent of NPS, data is collected by the European Union Drugs Agency (EUDA) in close contact with Europol from various sources to allow timely detection and response to emerging trends. The EUDA issued an early warning for 3,4-methylenedioxyphenmetrazine (MDPM, also known as 3,4-MDPM, 3-MDPM, 2-(1,3-benzodioxol-5-yl)-3-methylmorpholine) in April 2024 (EUDA 2024a). MDPM was first seized in Europe in June 2023 (EUDA 2024b) and its structure (Fig. 1) is related to the stimulants amphetamine, 3,4-methylenedioxymethamphetamine (MDMA) and phenmetrazine and is thus expected to have similar effects. To date, no information is available regarding the toxicokinetic properties of MDPM and no urinary biomarker of MDPM are available to serve as screening targets in toxicological analysis and doping control. Therefore, this study should provide such data on its toxicokinetics. This included in vitro plasma protein binding (PPB), in vitro half-life and in vitro metabolic fate by pooled human liver S9 fraction (pHLS9), pooled human liver microsomes (pHLM), or HepaRG cells. Furthermore, data on the isozyme specific biotransformation and possible cytochrome P450 (CYP) inhibition of MDPM should be provided.

## Materials and methods

### Chemicals and reagents

MDPM was provided by the University of Applied Sciences Kaiserslautern, Germany. Identity was confirmed with a purity > 98% using infrared spectroscopy and HPLC–MS. A 1 mg/mL stock solution of MDPM was prepared in methanol. Alpha-naphthoflavone, amodiaquine 2HCl, bupropion HCl, diclofenac, nicotinamide adenine dinucleotide phosphate (NADP^+^), omeprazole, phenacetin, sulfaphenazole, and trimethoprim were obtained from Sigma-Aldrich (Steinheim, Germany), dextromethorphan from Roche (Grenzach, Germany), fluconazole from Pfizer (Berlin, Germany), quinidine from Chininfabrik Buchler (Braunschweig, Germany), and testosterone from Fluka (Neu-Ulm, Germany). 3′-Phosphoadenosine-5′-phosphosulfate (PAPS), acetylcarnitine, acetyl coenzyme A (AcCoA), carnitine acetyltransferase, dimethyl sulfoxide (DMSO), dipotassium hydrogen phosphate (K_2_HPO_4_), dithiothreitol (DTT), isocitrate, isocitrate dehydrogenase, magnesium chloride (MgCl_2_), potassium dihydrogen phosphate (KH_2_PO_4_), reduced glutathione (GSH), S-(5′-adenosyl)-l-methionine (SAM), superoxide dismutase, tris HCl, verapamil HCl, and two-chambered Centrifree devices were purchased from Merck KGaA (Darmstadt, Germany). UDP-glucuronic acid 25 mM (UGT reaction mixture solution A), 250 mM Tris HCl, 40 mM MgCl_2_, and 125 μg/mL alamethicin (UGT reaction mixture solution B) were obtained from Corning (Amsterdam, Netherlands). Water was purified using a Millipore filtration unit. Pooled human liver microsomes (20 mg microsomal protein/mL, 25 donors), pooled human liver S9 fraction (20 mg microsomal protein/mL, 30 donors), baculovirus-infected insect cell microsomes (Supersomes) containing human cDNA-expressed CYP isoforms CYP1A2, CYP2B6, CYP2C8, CYP2C19, CYP2D6, CYP3A4, CYP3A5 (1 nmol/mL); CYP2A6, CYP2C9, and CYP2E1 (2 nmol/mL), and flavin-containing monooxygenase 3 (FMO3, 5 mg/mL) were purchased from Discovery Life Sciences (Huntsville, LA, USA). All enzyme containing preparations were thawed at 37 °C after delivery, aliquoted, snap-frozen in liquid nitrogen and stored at − 80 °C until use. Trimipramine-d_3_ was from LGC (Wesel, Germany). Williams E medium, HPRG670 supplement, GlutaMAX, penicillin, streptomycin, cryopreserved and differentiated HepaRG cells, and type I collagen-coated 96-well plates were purchased from Life Invitrogen (Darmstadt, Germany). Sertraline, acetonitrile, methanol, formic acid (LC–MS grade each), ammonium formate (analytical grade), and all other reagents and chemicals (analytical grade) were from VWR (Darmstadt, Germany). 96-Well plates were purchased from Sarstedt (Nümbrecht, Germany).

### Plasma protein binding

PPB of MDPM was investigated based on published procedures with minor adjustments (Gampfer et al. 2020), (Mardal et al. 2016). A volume of 450 µL fresh human blood plasma was spiked with 50 µL of a 5 µM methanolic solution of MDPM. The mixture was incubated for 30 min at 37 °C. Plasma aliquots of 100 µL and 400 µL were transferred to a new reaction tube (global approach, GA) and onto two-chambered Centrifree devices from Merck (Darmstadt, Germany), respectively. The Centrifree devices were centrifuged for 40 min and 1600×*g* to obtain 100 µL of ultrafiltrate (UF). UF and GA were precipitated using 50 µL acetonitrile (− 20 °C). The mixture was vortexed, cooled for 30 min at − 20 °C, and centrifuged for 3 min at 18,407×*g*. A volume of 100 µL of the supernatant was transferred into an autosampler vial and 10 µL were injected onto the LC-HRMS/MS system. Experiments were done in triplicate. Fraction unbound (*f*_*u*_) and PPB was determined by comparing the area ratios of MDPM and trimipramine-d_3_ in the UF and GA using the following equations:

1 $${f}_{u}=\frac{\text{peak area ratio}\left(\frac{{\text{MDPM}}_{\text{UF}}}{{\text{IS}}_{\text{UF}}}\right)}{\text{peak area ratio}\left(\frac{{\text{MDPM}}_{\text{GA}}}{{\text{IS}}_{\text{GA}}}\right)}$$

2 $$\text{PPB }[{\%}]=\left(1-{f}_{u}\right)*100$$

Lipophilicity (logP) of all compounds were calculated with ChemDraw version 23.1.1 (PerkinElmer, Waltham, MA, USA).

### Incubations using pooled human liver microsomes

Incubations with pHLM were in accordance with previous publications and minor modifications (Richter et al. 2017). First, 2.5 mM isocitrate, 0.8 U/mL isocitrate dehydrogenase, 100 U/mL superoxide dismutase, 0.6 mM NADP^+^, 2.5 mM Mg^2+^ and pHLM (1 mg microsomal protein/mL) were preincubated for 10 min at 37 °C. Reaction was started by adding 25 µM MDPM or 25 µM verapamil (positive control). Substances were incubated for 60 and 120 min at 37 °C. All concentrations are final concentrations, and incubations were done in duplicates. 50 µL aliquots were transferred to a new reaction tube at both timepoints. The reaction was stopped by adding 30 µL acetonitrile (− 20 °C) with 2.5 µM trimipramine-d_3_ as an internal standard. Negative control samples without enzymes and blank samples without substrates were performed to identify not metabolically formed compounds and to confirm the absence of interfering compounds. Organic solvent in the incubation mixture was below 1% (Chauret et al. 1998). Afterwards the samples were vortexed and centrifuged for 2 min at 18,407×*g*. 70 µL of the supernatant were transferred to autosampler vials and 10 µL were injected onto the LC-HRMS/MS system.

### Incubations using pooled human liver S9 fraction

Incubations with pHLS9 were performed in accordance with previous publications and minor adjustments (Richter et al. 2017). First, 25 μg/mL alamethicin (UGT reaction mix B), pHLS9 (2 mg microsomal protein/mL), 0.1 mM AcCoA, 2.3 mM acetylcarnitine, 8 U/mL carnitine acetyltransferase, 2.5 mM isocitrate, 0.8 U/mL isocitrate dehydrogenase, 100 U/mL superoxide dismutase, 0.6 mM NADP^+^ and 2.5 mM Mg^2+^ were preincubated for 10 min at 37 °C. Then, 2.5 mM UDP-glucuronic acid (UGT reaction mix A), 40 µM PAPS, 1.2 mM SAM, 1 mM DTT and 10 mM GSH were added. The reaction was started by adding the respective substrate MDPM, MDMA, or quetiapine (25 µM each). MDMA and quetiapine were incubated as positive controls. Negative control samples without enzymes and blank samples without substrates were incubated to identify not metabolically formed compounds and to confirm the absence of interfering compounds. Incubations were done in duplicate and organic solvent in the incubation mixture was below 1% (Chauret et al. 1998). Aliquots (50 µL) were transferred to a new reaction tube after 60 and 360 min. The reaction was stopped with 30 µL acetonitrile (− 20 °C) containing 2.5 µM trimipramine-d_3_ as an internal standard. Samples were vortexed, stored at − 20 °C for 30 min, and centrifuged for 2 min at 18,407×*g*. 70 µL of the supernatant were transferred to autosampler vials and 10 µL were injected onto the LC-HRMS/MS system.

Incubations to investigate in vitro half-life were performed under the same incubation conditions as described above and done in duplicate. Thirty µL aliquots were taken after 0, 15, 30, 45, 60, 75, 90 and 180 min and were terminated with 20 µL acetonitrile (− 20 °C) containing 2.5 µM trimipramine-d_3_ as an internal standard. 40 µL of the supernatant were transferred to autosampler vials and 10 µL were injected onto the LC-HRMS/MS system. A *t*-test was performed to compare absolute peak areas of the incubation group and the control group at 0 min. The following parameters were used: unpaired; two-tailed; significance level, 0.05; confidence intervals, 99%.

### Incubations using HepaRG cells

Cell incubations were performed in a monolayer assay as previously described (Richter et al. 2019) and in accordance with the manufacturer’s instructions. HepaRG cells were thawed and resuspended in 13.5 mL (~ 740,000 cells/mL) thaw and seed medium at 37 °C. Thaw and seed medium consisted of Williams E medium, 100 U/mL penicillin, 100 µg/mL streptomycin, GlutaMAX and HPRG670 supplement. Cells were handled under sterile conditions using a laminar flow bench class II (Thermo Scientific, TF, Schwerte, Germany). Aliquots of 100 µL cell suspension were seeded on collagen-coated 96-well plates (~ 74,000 cells/well). Evaporation was minimized by filling the outer wells with 100 µL thaw and seed medium. Cells were preincubated for 4 h in an incubator (Binder, Tuttlingen, Germany) at 37 °C, 95% air humidity, and 5% CO_2_. After preincubation, 50 µL of the thaw and seed medium was replaced with 50 µL MDPM solution (25 µM and 250 µM final concentrations), followed by an incubation for 24 h at 37 °C, 95% air humidity, and 5% CO_2_ atmosphere. MDMA or quetiapine (25 µM and 250 µM final concentrations, respectively) were incubated as positive controls. All incubations were done in triplicates. Each well contained 0.5% (*v/v*) DMSO. After incubation, 50 µL of the medium supernatant was precipitated in a new reaction tube with 30 µL acetonitrile (− 20 °C) containing 2.5 µM trimipramine-d_3_ as an internal standard. A negative control sample without HepaRG cells and a blank sample without substrate were performed to identify not metabolically formed compounds and to confirm the absence of interfering compounds. The mixture was vortexed, cooled for 30 min at − 20 °C, and centrifuged for 3 min at 18,407×*g*. 70 µL of the supernatant was transferred into an autosampler vial and 10 µL were injected onto the LC-HRMS/MS system.

### Monooxygenase mapping

As described elsewhere (Wagmann et al. 2016) and with minor modifications, MDPM (25 µM) was incubated with 2.5 mM isocitrate, 0.8 U/mL isocitrate dehydrogenase, 100 U/mL superoxide dismutase, 0.6 mM NADP^+^, 2.5 mM Mg^2+^ and CYP1A2, CYP2A6, CYP2B6, CYP2C8, CYP2C9, CYP2C19, CYP2D6, CYP2E1, CYP3A4, CYP3A5 (50 pmol/mL each), or FMO3 (0.25 mg protein/mL) for 90 and 270 min at 37 °C. For incubations with CYP2A6 or CYP2C9, phosphate buffer was replaced with 90 mM Tris buffer, according to the manufacturer’s guideline. Thirty µL aliquots were taken at both timepoints. The reaction was stopped using 20 µL acetonitrile (− 20 °C) with 2.5 µM trimipramine-d_3_ as an internal standard. All incubations were done in duplicates and concentrations are final. Verapamil (25 µM) was incubated as a positive control. Negative control samples without enzymes and blank samples without substrate were incubated to identify not metabolically formed compounds and to confirm the absence of interfering compounds. The amount of organic solvent in the incubation mixture was below 1% (Chauret et al. 1998). Afterwards the samples were vortexed and centrifuged for 2 min at 18,407×*g*. 40 µL of the supernatant were transferred to autosampler vials and 10 µL were injected onto the LC-HRMS/MS system.

### CYP inhibition studies

A modified dual-cocktail based method (Dinger et al. 2014b) was used. Substrate cocktail A consisted of 8.9 µM dextromethorphan (CYP2D6), 86 µM testosterone (CYP3A4), 3.5 µM diclofenac (CYP2C9), and 12 µM phenacetin (CYP1A2). Substrate cocktail B included 30 µM bupropion (CYP2B6), 2 µM amodiaquine (CYP2C8) and 21 µM omeprazole (CYP2C19). Substrate concentrations were near their respective Michaelis–Menten constant (K_m_) values (Dinger et al. 2014a) and all concentrations are final. Enzyme inhibition was investigated using a positive control, a test group, a control group, an interference group, and a negative control. In the test group, specific inhibitors were replaced by MDPM (20 µM). Instead of inhibitors, phosphate buffer was used in the control group. Enzymes were replaced with phosphate buffer in the negative control group. The interference group was incubated like the control group, but the reaction was stopped with 20 µM MDPM in acetonitrile, also containing 2.5 µM trimipramine-d_3_. All groups were incubated with a NADPH-regenerating system, consisting of 5 mM isocitrate, 2 U/mL isocitrate dehydrogenase, 5 mM Mg^2+^, and 1.2 mM NADP^+^. Further, substrate cocktail A or B, 90 mM phosphate buffer (pH 7.4), and 200 U/mL superoxide dismutase were added to each group. The positive control included specific inhibitors (20 µM each, except for 100 µM trimethoprim) of the respective investigated enzymes (cocktail A or B). Specific inhibitors in cocktail A were quinidine (CYP2D6), verapamil (CYP3A4), alpha-naphthoflavone (CYP1A2), and sulfaphenazole (CYP2C9). In cocktail B, sertraline (CYP2B6), trimethoprim (CYP2C8), and fluconazole (CYP2C19) were used. All groups were preincubated for 10 min at 37 °C. Then, pHLM (0.4 mg microsomal protein/mL) was added to each group except the negative control. Incubations were done for 15 min at 37 °C. Therefore, 50 µL of acetonitrile (− 20 °C) containing 2.5 µM trimipramine-d_3_ as internal standard was added. Incubations were done in triplicates and organic solvent in the incubation mixture was below 1% (Chauret et al. 1998). Afterwards the samples were vortexed and centrifuged for 2 min at 18,407×*g*. 70 µL of the supernatant were transferred to autosampler vials and 10 µL were injected onto the LC-HRMS/MS system. Enzyme inhibition was evaluated by metabolite formation. A *t*-test was performed to investigate significant inhibition in the positive control and test group compared to the control group (rejection of null hypothesis) or to prove similarity in results in the interference group and negative control (acceptance of null hypothesis) compared to the control group. The following settings were used: unpaired; one-tailed; significance level, 0.05; confidence intervals, 99%.

### LC-HRMS/MS apparatus and conditions

A TF (Dreieich, Germany) Dionex UltiMate 3000 RS pump consisting of a degasser, a quaternary pump, and an HTC prep and load (PAL) autosampler, coupled to a TF Q Exactive system equipped with a heated electrospray ionization (HESI)-II source were used. A mass calibration was done according to the manufacturer's recommendations using external mass calibration prior to analysis. Gradient elution was performed according to a previous study (Helfer et al. 2015) on a TF Accucore PhenylHexyl column (100 mm × 2.1 mm, 2.6 μm) with a 2 mM aqueous ammonium formate solution containing 0.1% (*v/v*) formic acid (pH 3, eluent A) and 2 mM ammonium formate solution in acetonitrile/methanol (50:50, *v/v*) containing 0.1% (*v/v*) formic acid, and 1% (*v/v*) water (eluent B). The gradient was stepped as follows: 0–2.5 min hold 99% A, 2.5–8 min to 1% A, 8–9.5 min hold 1% A, and 9.5–11.5 min hold 99% A. Initial flow rate from 0–9.5 min was 500 μL/min and final flow rate was 800 μL/min from 9.5–11 min. Injection volume was 10 μL for every sample. Mass spectrometry was performed using full scan data and a subsequent data-dependent acquisition (DDA) with priority to mass-to-charge ratios (*m/z*) of parent compounds and their expected metabolites. The inclusion list contained *m/z* values of likely formed metabolites such as *O*-demethylenyl and hydroxy metabolites (phase I) as well as sulfates, glucuronides, methoxy metabolites and combinations thereof (phase II). Chemdraw 23.1.1 was used to draw structures of expected metabolites and for exact mass calculations. TF Xcalibur Qual Browser software version 4.6 (Dreieich, Germany) was used for data handling. Mass deviations of the parent compound were accepted up to 5 ppm. Plasma protein binding samples were measured only in positive ionization mode, all other samples were measured in positive and negative ionization mode.

Instrument settings, data generation settings*,* and data evaluation settings are shown in Table S1 in the supporting information.

## Results

### In vitro plasma protein binding and in vitro half-life

In vitro PPB of MDPM was calculated to be 32% (Table 1**,** calculated logP = 1.3). In vitro half-life was determined by measuring substrate depletion. The natural logarithm of the absolute peak areas of MDPM was plotted against time, as shown in Figure S1 of the supporting information. All linearity criteria were met, such as an enzyme concentration of 1 mg/mL (final) a low substrate concentration (Baranczewski et al. 2006), and incubation time. Ideally, the concentration should be below K_m_ value for known compounds, or in the case of MDPM as low as possible. Half-life was estimated to be > 180 min, which indicated low turnover. Additional toxicokinetic data can be found in Table 1.

**Table 1:** Toxicokinetic data of 3,4-Methylenedioxyphenmetrazine (MDPM) including the unbound fraction (*f*_*u*_), plasma protein binding (PPB), lipophilicity (logP) and half-life (*t*_1/2_)

Parameter	Value
*f*_*u*_	0.68
PPB	32%
logP	1.3
*t*_1/2_	> 180 min

### Mass spectra, in vitro metabolites formed by pHLM, pHLS9 and HepaRG cells, and monooxygenase mapping

**Fig. 2:**
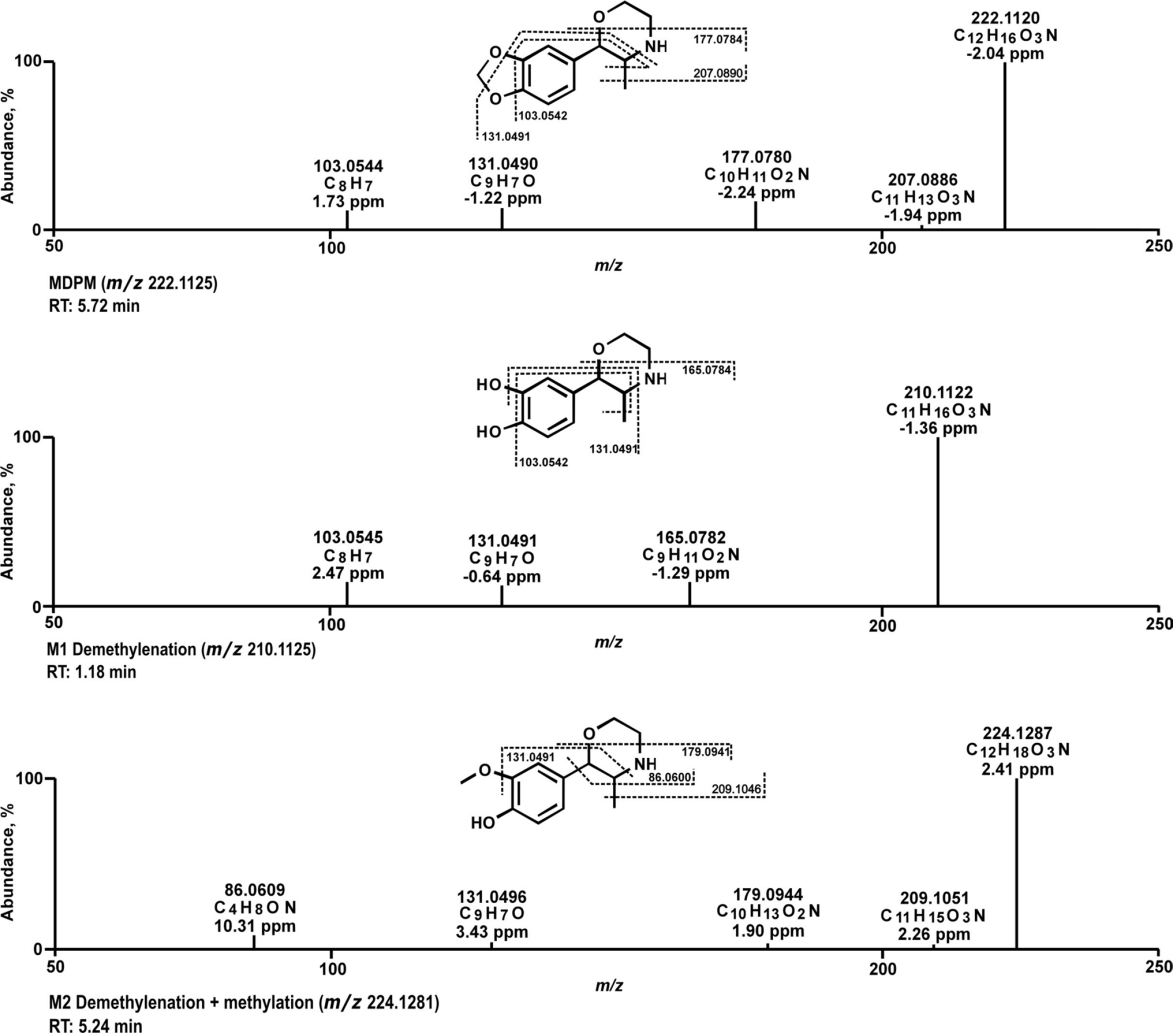
MS^2^ spectra of 3,4-Methylenedioxyphenmetrazine (MDPM) and the proposed phase I and phase II metabolites, sorted by metabolic reaction phase. RT: retention time

All mass spectra in the following are presented using the exact masses and all metabolites are considered as tentative, as there were no reference standards available. Measured (accurate) and calculated (exact) *m/z*, elemental composition, mass error and retention time (RT) of precursor ions (PI) and fragment ions (FI) are listed in Table S2 in the supporting information. MS^2^ spectra of MDPM and its metabolites identified in vitro are depicted in Fig. 2. Within the spectrum of MDPM, the PI at *m/z* 222.1125 was observed as the base peak. Elimination of the morpholine ring methyl group resulted in a radical FI at *m/z* 207.0890. The radical FI at *m/z* 177.0784 was formed by elimination of C2H4O next to the secondary amine, and in benzylic position with loss of water under formation of a double bond. Further elimination of CO from the methylenedioxy group and loss of ammonia led to the FI at *m/z* 131.0491. Finally, an ethylbenzene FI at *m/z* 103.0542 was formed after homolytic cleavage of the methyl group and a loss of water at the aromatic system. One phase I and one phase II metabolite were tentatively identified in vitro. *M1* with PI at *m/z* 210.1125 was formed by demethylenation. Fragmentation pattern was similar to the parent compound with FI at *m/z* 131.0491 and at *m/z* 103.0542 except of the FI at *m/z* 165.0784. This FI corresponded to the FI at *m/z* 177.0784 shifted by a carbon atom. *M2* with PI at *m/z* 224.1281 was the result of the demethylenation followed by *O*-methylation. The FI at *m/z* 131.0491 was identical to the parent compound, and the FIs at *m/z* 209.1046 and *m/z* 179.0941 corresponded to the FIs at *m/z* 207.0890 and 177.0784 of the parent compound respectively, shifted by two hydrogen atoms. *M2* further showed a fragmentation of the morpholine ring at *m/z* 86.0600. *M1* was found in pHLM incubations after 60 and 120 min but could not be identified in pHLS9 and HepaRG incubations, most likely due to rapid *O*-methylation (*M2*) in both models. *M2* was found in both pHLS9 and HepaRG at 60 and 360 min. HepaRG incubations with 250 µM MDPM resulted in a more abundant signal of the phase II metabolite than with 25 µM. The demethylenyl-methyl metabolite could only be detected in two of three HepaRG incubations with 25 µM MDPM. No metabolites were detected in blank samples (no substrate) or negative control (no enzymes). Both positive controls MDMA and quetiapine, formed characteristic phase I and phase II metabolites. MDMA, as a structurally related substance of MDPM, formed the *N*-dealkylated metabolite, as well as demethylenyl-methyl MDMA. No other phase II metabolites, such as the glucuronide described in literature (Steuer et al. 2016), could be identified. Demethylenyl-MDPM (*M1*) was only found in CYP2D6 incubations and results were in line with the positive control (pHLM).

### CYP inhibition potential

**Fig. 3:**
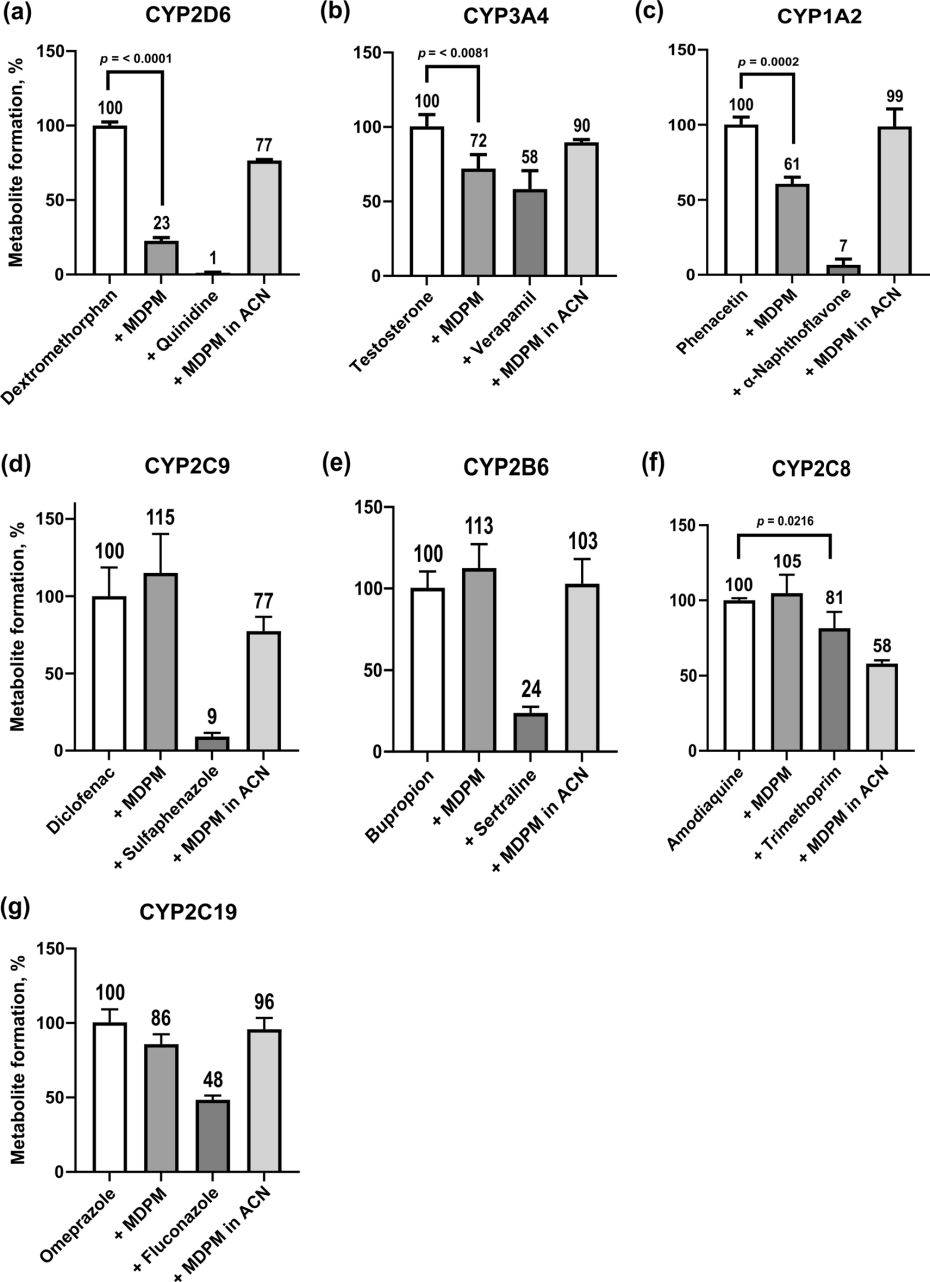
Inhibition potential of MDPM on the metabolism of selected substrates by **a** CYP2D6 (dextromethorphan), **b** CYP3A4 (testosterone), **c** CYP1A2 (phenacetin), **d** CYP2C9 (diclofenac), **e** CYP2B6 (bupropion), **f** CYP2C8 (amodiaquine), **g** CYP2C19 (omeprazole) measured as reduction of relative enzyme activity in percent. First bar: control group (no inhibitor), second bar: test group (MDPM as test inhibitor), third bar: positive control group (specific inhibitor), fourth bar: interference group (control group, stopped with MDPM in acetonitrile)

Inhibition results of all CYP enzymes are summarized in Fig. 3. No metabolite formation was observed in the negative control group (no enzymes), which is not shown in Fig. 3. CYP inhibition of MDPM was compared to a control group without inhibitor, where metabolite formation was set to 100%. Significant inhibition was observed in all positive control groups (a–g). As in other studies a maximum of 40% of CYP2C8 activity was inhibited using 100 µM trimethoprim (Niemi et al. 2004), a concentration of 100 µM instead of 20 µM was used. However, only an inhibition of ~ 20% was observed but still significant (f). Interference groups were used to control signal enhancement or suppression between MDPM and the monitored metabolites due to the chosen cocktail approach. Thereby, false positive or negative results could be minimized. The interference group of CYP2D6 (a) and CYP2C8 (f) showed a low but significant signal suppression. Signal enhancement was not observed for any interference group. A significant decrease in metabolite formation, also to the interference group, of dextromethorphan by MDPM (77% decrease in CYP2D6 activity) was observed in the test group (a). The model inhibitor quinidine (positive control) also showed a significant inhibition of 99%. Further, MDPM showed moderate but significant inhibition of CYP3A4 ((b), 28% decrease in enzyme activity) and CYP1A2 ((c), 39% decrease in enzyme activity), respectively. MDPM showed no significant inhibition for CYP2C9, CYP2B6, CYP2C8, and CYP2C19 (d–g).

## Discussion

PPB was determined using regenerated cellulose membranes, which were shown to eliminate the effect of non-specific binding (Heinze and Holzgrabe 2006; Lier et al. 2024). Data on PPB is crucial as it influences drug distribution, elimination and only the unbound fraction of a compound can usually exert effects, cause side effects, and potentially lead to toxicity. The PPB of MDPM (32%, logP = 1.3) is in range of that of phenmetrazine (20%) (Franksson and Anggard 1970) with a calculated logP = 1.5, of MDMA (43–51%) (Wan Aasim et al. 2017) with a calculated logP = 2.0, and of amphetamine (20–30%) (Franksson and Anggard 1970; Losacker et al. 2021), calculated logP = 1.7. These data show that low protein binding (< 50%) is common for many amphetamine-like stimulants. Lipophilicity and PPB often show high correlation, and comparatively low to moderate lipophilicity (logP 1–3), as shown for MDPM, is consistent with a low level of PPB (Laznicek and Laznickova 1995). Changes in the bound fraction only have high impact on the unbound fraction if the plasma protein binding exceeds 90% (McLeod and He 2012). The in vitro half-life of MDPM was estimated to be > 180 min, which indicated low turnover. Similar results can be found in literature for MDMA and other methylenedioxy-compounds, showing an in vitro half-life of over 150 min (Alberto-Silva et al. 2024). Compared to pHLM, the phase I enzyme activity in pHLS9 is relatively low, which could explain low turnover. However, linearity is achieved in both systems and pHLS9 offers the advantage to also detect directly formed phase 2 metabolites. Rapid metabolism and non-metabolic degradation before initial sampling could be excluded, since the *t*-test showed no significant difference between the incubation sample and the control group at min 0. In vitro metabolism data by e.g., pHLS9 or HepaRG cells are often in accordance with metabolites found in human samples (Wagmann et al. 2020). Therefore, pHLS9 and HepaRG cells were used in addition to pHLM, which typically shows a higher CYP enzyme activity compared to pHLS9. Metabolites were tentatively identified by first comparing experimental masses with exact masses of PI of MDPM and its expected metabolites. Subsequently, fragmentation patterns in DDA mode were compared between MDPM and its potential metabolites. Based on the in vitro data, the demethylenyl-methyl metabolite of MDPM next to the parent compound can be recommended as analytical urine screening target. In vivo data from e.g., rats or human would be beneficial to confirm suggested biomarkers. Monooxygenases activity screening provides information on enzymes involved in the phase I metabolism. This could enable a prediction of possible drug-drug interactions. In comparison, MDMA *O*-demethylenation is also mainly catalyzed by CYP2D6 (Tucker et al. 1994). Exclusive metabolism of a dominant step by one CYP enzyme in the metabolism of xenobiotics is at risk to be affected by interactions such as CYP inhibition. This is the case for MDPM as *O*-demethylenation is catalyzed exclusively by CYP2D6. Not only inhibition of CYP2D6 but also polymorphisms of CYP2D6 could also affect the metabolic elimination of MDPM. Inhibition potential of MDPM was investigated towards the activity of seven selected CYP isozymes, accounting for the majority of xenobiotic metabolism in humans (Tseng et al. 2024), (Wojcikowski et al. 2020). First due to the low metabolic turnover rate of MDPM and second due to the known inhibition potential of other methylenedioxy compounds (Meyer et al. 2009). CYP2D6 inhibition is also known for other methylenedioxy-drugs and amphetamine-like stimulants (Meyer et al. 2009), (Dinger et al. 2016). MDMA, e.g., is a mechanism-based inhibitor of CYP2D6, which may lead to a higher risk for overdosing and toxic events in recreational consumers (de la Torre et al. 2012). Although it is possible that MDPM competitively inhibited dextromethorphan degradation, a mechanism-based inhibition might also be assumed. In that case, MDPM would inhibit its own degradation via CYP2D6. Given the significant in vitro inhibition of CYP2D6 by MDPM, toxic effects and overdosing may be observed in vivo after MDPM consumption with other CYP2D6 substrates. Blood plasma concentrations of simultaneously consumed xenobiotics such as NPS metabolized via CYP3A4 or CYP1A2 may also be increased. Potentially toxic drug-drug interactions should be considered after consumption of MDPM.

## Conclusions

The in vitro toxicokinetics of MDPM were investigated, including plasma protein binding, half-life, metabolism, monooxygenases activity screening, and CYP inhibition. MDPM showed a low plasma protein binding, which was in line with literature data of similar compounds and is thus not expected to be of major interest concerning drug-drug interactions. Further, high in vitro half-life indicates low turnover, which is most likely due to strong inhibition of CYP2D6 by MDPM, of which MDPM is also a substrate. Metabolism was limited to demethylenation and *O*-methylation in both pHLS9 and HepaRG incubations. Monooxygenases activity screening revealed that the demethylenyl reaction is solely CYP2D6-dependent. Further, moderate inhibition of CYP3A4 and CYP1A2 was observed. Drug-drug interactions should be considered if other drugs (of abuse) are co-consumed which are metabolized by these isozymes. Certain polymorphisms of CYP2D6, especially in combination with drug-drug interactions or environmental factors, increase the likelihood of an intoxication. Recommended toxicological routine screening targets are the parent compound next to the demethylenyl-methyl metabolite.

## Supplementary Information

Below is the link to the electronic supplementary material.

Supplementary file1 (PDF 75 KB)

## Data Availability

Data might be made available upon reasonable request from the authors.
