# Supplementary material for: Toxicokinetics and analytical toxicology of the phenmetrazine-derived new psychoactive substance 3,4-methylenedioxyphenmetrazine studied by means of in vitro systems
Source: Arch Toxicol. 2025 Feb 4;99(4):1407–16. doi: 10.1007/s00204-025-03965-w (PMC11968506; doi:10.1007/s00204-025-03965-w)
Supplement: Supplementary file 1 — Supplementary file1 (PDF 75 KB) [file 204_2025_3965_MOESM1_ESM.pdf]

Electronic Supplementary Material

Toxicokinetics and analytical toxicology of the phenmetrazine-derived new psychoactive substance 3,4-methylenedioxyphenmetrazine studied by means of in vitro systems

Matthias D. Kroesen, Tanja M. Gampfer, Lea Wagmann, Markus R. Meyer*

Department of Experimental and Clinical Toxicology and Pharmacology, Institute of Experimental and Clinical Pharmacology and Toxicology, Center for Molecular Signaling (PZMS), Saarland University, Homburg, Germany

**Correspondence:* m.r.meyer@mx.uni-saarland.de

Table S1. Instrument setting for analysis using the TF Q Exactive system.

**HESI-II source conditions**	
Heater temperature	320 °C
Ion transfer capillary temperature	320 °C
Spray voltage	4.0 kV
Ionization mode	Positive and negative
Sheath gas	60 arbitrary units (AU)
Auxiliary gas	10 AU
Sweep gas	0 AU
S-lens RF level	50.0
**Full scan data acquisition**	
Resolution	35,000
Microscans	1
Automatic gain control (AGC) target	1e6
Maximum injection time (IT)	120 ms
Scan range	*m/z* 50-750
**Settings for DDA mode plus inclusion list (MDPM and metabolites)**	
Option "pick others"	Enabled
Dynamic exclusion	0.1 s
Resolution	17,500
Microscans	1
Isolation window	1.0 *m/z*
Loop count	5
AGC target	2e5
Maximum IT	250 ms
high collision dissociation cell with stepped normalized collision energy	17.5, 35.0, 52.5
Exclude isotopes	On
Spectrum data type	Profile
Underfill ratio	1 %
**Peak integration**	
Peak detection algorithm	INCOS
Baseline window	40
Area noise factor	5
Peak noise factor	10

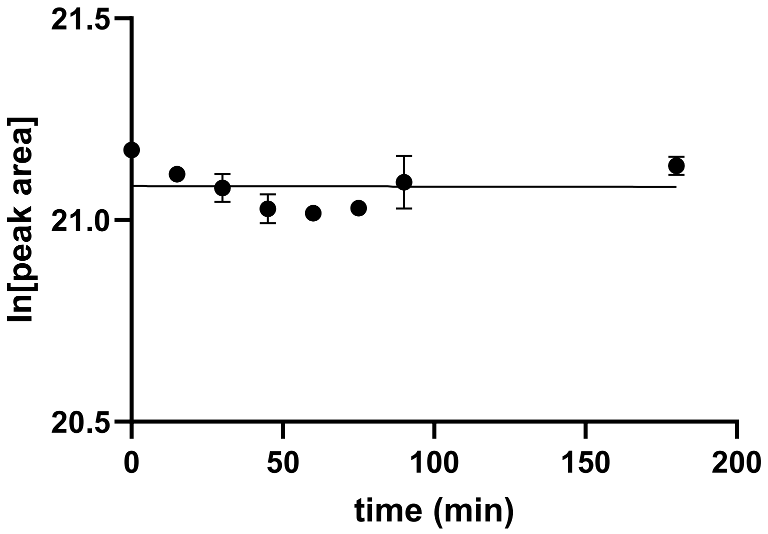

Figure S1

Metabolic stability of MDPM (25 µM) in pHLS9 (2 mg microsomal protein/mL). Incubation time (min) was plotted against the natural logarithm of full scan peak areas. Data represents SD from two independent incubations (*n* = 2).

Table S2. List of 3,4-Methylenedioxyphenmetrazine (MDPM) and its metabolites with precursor ion (PI) masses recorded in MS^1, characteristic fragment ions (FI) in MS^2, calculated exact masses, elemental composition, mass error, and retention time (RT). Metabolites are sorted according to their reaction phase.

Metabolite ID	Metabolic reaction	Measured masses of PI and characteristic FI, *m/z*	Calculated exact masses, *m/z*	Elemental composition	Mass error, ppm	RT, min
MDPM		PI at *m/z* 222.1120	222.1125	$C_{12}H_{16}O_3N$	-2.04	5.72
		FI at *m/z* 207.0886	207.0890	$C_{11}H_{13}O_3N$	-1.94	
		FI at *m/z* 177.0780	177.0784	$C_{10}H_{11}O_2N$	-2.24	
		FI at *m/z* 131.0490	131.0491	C_9H_7O	-1.22	
		FI at *m/z* 103.0544	103.0542	C_8H_7	1.73	
M1	Demethylenation	PI at *m/z* 210.1122	210.1125	$C_{11}H_{16}O_3N$	-1.36	1.18
		FI at *m/z* 165.0782	165.0784	$C_9H_{11}O_2N$	-1.29	
		FI at *m/z* 131.0491	131.0491	C_9H_7O	-0.64	
		FI at *m/z* 103.0545	103.0542	C_8H_7	2.47	
M2	*O*-Methylation	PI at *m/z* 224.1287	224.1281	$C_{12}H_{18}O_3N$	2.41	5.24
		FI at *m/z* 209.1051	209.1046	$C_{11}H_{15}O_3N$	2.26	
		FI at *m/z* 179.0944	179.0941	$C_{10}H_{13}O_2N$	1.90	
		FI at *m/z* 131.0496	131.0491	C_9H_7O	3.43	
		FI at *m/z* 86.0609	86.0600	C_4H_8ON	10.31	
